# A novel pharmacological mechanism of anti-cancer drugs that induce pyroptosis

**DOI:** 10.1007/s10787-023-01148-6

**Published:** 2023-03-03

**Authors:** Haohao Guo, Ziyang Wang, Runsheng Ma, Xin Chen, Hongqiang Li, Yifeng Tang, Gongbo Du, Yifei Zhang, Detao Yin

**Affiliations:** 1 Department of Thyroid Surgery, the First Affiliated Hospital of Zhengzhou University, Zhengzhou, 450052 Henan China; 2 Engineering Research Center of Multidisciplinary Diagnosis and Treatment of Thyroid Cancer of Henan Province, Zhengzhou, 450052 Henan China; 3 Key Medicine Laboratory of Thyroid Cancer of Henan Province, Zhengzhou, 450052 Henan China

**Keywords:** Pyroptosis, Pharmacology, Anti-cancer, Gasdermin family

## Abstract

Pyroptosis is an inflammasome-induced lytic form of programmed cell death, and its main effect involves the release of inflammatory mediators when a cell dies, resulting in an inflammatory response in the body. The key to pyroptosis is the cleavage of GSDMD or other gasdermin families. Some drugs can cause cleavage GSDMD or other gasdermin members cause pyroptosis and suppress cancer growth and development. This review explores several drugs that may induce pyroptosis, thereby contributing to tumor treatment. Pyroptosis-inducing drugs, such as arsenic, platinum, and doxorubicin, were used originally in cancer treatment. Other pyroptosis-inducing drugs, such as metformin, dihydroartemisinin, and famotidine, were used to control blood glucose, treat malaria, and regulate blood lipid levels and are effective tumor treatments. By summarizing drug mechanisms, we provide a valuable basis for treating cancers by inducing pyroptosis. In future, the use of these drugs may contribute to new clinical treatments.

## Introduction

The term “pyroptosis” is derived from the Greek root “pyro”, meaning fire or fever, and “ptosis”, meaning falling. It was coined to describe pro-inflammatory programmed cell death and a generally intrinsic immune response mechanism in vertebrates (Cookson & Brennan 2001; Galluzzi et al. 2018; Jorgensen & Miao 2015). In 2002, inflammatory bodies were initially thought to activate inflammatory caspases in response to pro-IL-1β/IL-18, inducing pyroptosis (Martinon et al. 2002).

Pyroptosis is regulated mostly by the gasdermin family, which consists of six members, namely, gasdermin A–E and DFNB59; most gasdermin proteins exhibit pore-forming ability (C. Rogers et al. 2017; Y. Wang et al. 2017). Except for DFNB59, which shows a divergent structure and short C-terminal domain, other gasdermin family members are composed of similar domains and overall structure (Kovacs and Miao 2017; Corey Rogers et al. 2017). The pore-forming domain (PFD) and repression domain (RD) of gasdermin proteins are similar, but the sequences that connect them differ (S. B. Kovacs & E. A. Miao, 2017; Kuang et al. 2017; Z. Liu et al. 2019). Each gasdermin family member shares an N-terminal domain and binds to acidic lipids, including phosphatidic acid (PA), phosphatidylinositol phosphate (PIP), cardiolipin, and phosphatidylserine (PS), forming pores comprising 16 symmetrical pre-polymers within the plasma membrane. Among gasdermin proteins, GSDMD and GSDME are activated and function with superior characteristics; however, the interaction of intramolecular fragments in the C-/N-terminal prevents the activated N-terminal domain from causing pyroptosis (Ding et al. 2016; X. Liu et al. 2016). GSDMD was the first gasdermin protein to be discovered, and it plays a vital role in pyroptosis (He et al. 2015; Kayagaki et al. 2015).

**Fig. 1:**
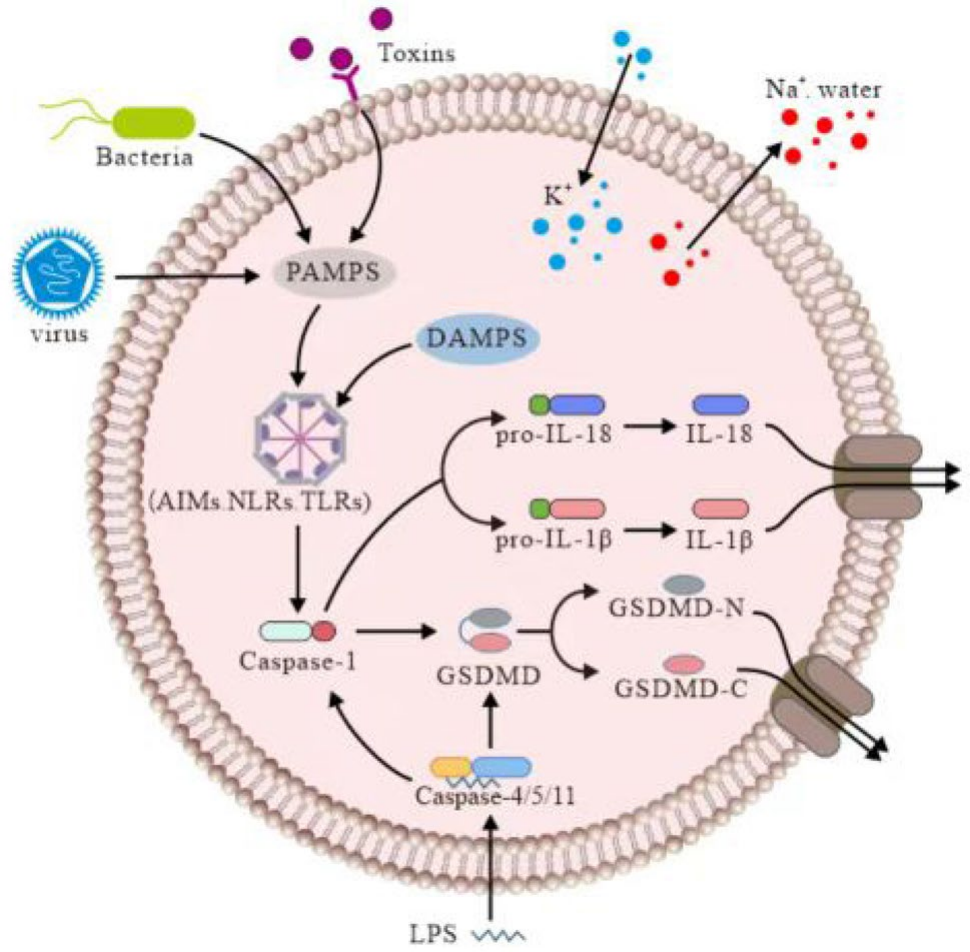
Pyroptosis contains classical inflammasome pathway and non-classical inflammasome pathways. Classical inflammasome pathway consists of DAMPs or PAMPs recognition by PRRs, which can be activated by ATP, bacteria, and viruses and trigger the NLR family pyrin domain containing 3 (NLRP3) inflammasome. Non-classical inflammasome pathways, initiated through caspase-4/5 and caspase-11 (mouse). NLRP3 activating and releasing the N-terminal domain of GSDMD (GSDMD-N), caspase-1 convert IL-1β/IL-18 precursors into mature IL-1β/IL-18. These factors are released via GSDMD-N-formed necrotic membrane pores

The inflammasome is a multi-molecular complex containing pattern-recognition receptors (PRRs) and induces pyroptosis upon activation PRRs recognize pathogen-associated molecular patterns (PAMPs) of invasive pathogens and damage-related molecular patterns (DAMPs) within the endogenous pathogens, directly eliminating intracellular bacterial species by the pore-mediated intracellular traps as well as destroying the pathogen replication niche (Burdette et al. 2021; Guo et al. 2015; Lamkanfi & Dixit 2017; Shi et al. 2017). The PRR family consists of several members, mainly including toll-like receptors (TLRs), retinoic acid-induced gene I (RIG-I)-like receptors (RLRs) and nucleotide oligomerization domain (NOD)-like receptors (NLRs) (Fang et al. 2020; Sborgi et al. 2016; L. Wang et al. 2021a, b). When PRRs recognize PAMPs or DAMPs, they induce apoptosis-associated speck-like protein containing a CARD (ASC), which forms supramolecular aggregates that connect NLRs and caspase-1 and induces the activation of the classical inflammasome pathway and the non-classical inflammasome pathway (Fernandes-Alnemri et al. 2007; Galluzzi et al. 2018; Xu et al. 2018). Although great progress has been made in tumor treatment, cancer remains the second leading cause of mortality worldwide, second only to cardiovascular disease (L. Li et al. 2021a, b; Morrison et al. 2018). Therefore, identifying new strategies for tumor treatment is crucial (Fig. 1).

With further study, pyroptosis has been found to be closely related to several human diseases, particularly cancers (Ruan; Tan et al. 2021; Yu et al. 2021). It affects tumor pathogenesis via two mechanisms. First, various signaling pathways and inflammatory factors produced during tumor pyroptosis have been associated with carcinogenesis and chemoresistance. Second, pyroptosis inhibits cancerogenesis and development. Several researches have indicated an association between pyroptosis and tumors. Therefore, pyroptosis can be included in various tumor treatment methods to inhibit cancer cell growth, invasion, and migration (Massagué & Obenauf 2016; Xia et al. 2019). This article reviews the potential of pyroptosis in tumor treatment.

## Mechanisms of pyroptosis

### Classical inflammasome pathway

The classical inflammasome pathway consists of DAMPs or PAMPs recognition by PRRs, which induce cytoplasmic signaling complex-mediated activation of the inflammasome as well as pyroptosis. Uric acid, adenosine triphosphate (ATP), bacteria, fungi, and viruses trigger the NLR family pyrin domain containing 3 (NLRP3) inflammasome. Although several PRRs are involved in the process, only a few PRRs can induce inflammasome assembly, which results in caspase-1 activation (Loveless et al. 2021). Different PAMPs and DAMPs, including fungal enzymes, bacterial lipopolysaccharides (LPS),extracellular ATP, viral RNA, melanin, reactive oxygen species (ROS), and amyloid-β plaques, can activate NLRP3 (Kelley et al. 2019). Moreover, NLRP3 activation induces lysosome damage, leading to K^+^ efflux as well as protease cathepsin B and lysosomal release of contents. Two proteases trigger the activation of the NLRP3 inflammasome (Alu et al. 2020). After activation, PRRs and inflammasomes interact with the ASC protein, which carries a C-terminal caspase recruitment domain, thereby activating caspase-1 by recruiting and cleaving pro-caspase-1. In addition to cutting GSDMD into the N-terminal structural domain of GSDMD (GSDMD-N), caspase-1 converts IL-1β/IL-18 precursors into mature IL-1β/IL-18 proteins. These factors are released via GSDMD-N-formed membrane pores during pyroptosis (Bergsbaken et al. 2009; Martinon et al. 2002; Van Gorp & Lamkanfi 2019) (Fig. 1).

### The non-classical inflammasome pathway

The non-classical inflammasome pathway, initiated through caspase-4/-5 and caspase-11 (mouse), contributes to cellular immunity against intracellular Gram-negative bacteria (Rathinam et al. 2019). Caspase, activated by LPS, does not directly activate IL-1β/IL-18 (Loveless et al. 2021). However, it cleaves GSDMD, leading to potassium efflux, NLRP3 activation, caspase-1 upregulation, neutrophil extracellular traps, and pyroptosis (Kayagaki et al. 2015; Shi et al. 2015; Silke & Vince 2017). Therefore, non-classical inflammasome links pyroptosis with necrosis to regulate neutrophil mortality stimulated by infectious pathogens (Burgener & Schroder 2020; Torii et al. 2016). The non-classical pathway has also been associated with mitochondrial dysfunction-induced metabolic diseases, including diseases related to mitochondrial DNA (mtDNA) and mitochondrial ROS release (Fig. 1).

### Pyroptosis-associated anti-cancer drugs

Several studies demonstrated that drugs inhibit tumor growth by inducing pyroptosis in tumor cells. The Food and Drug Administration has approved several clinical drugs, such as cisplatin, doxorubicin, and dihydroartemisinin, which induce pyroptosis in several cancer types. Because the number of cancer cases is steadily increasing worldwide, identifying drugs that inhibit cancer development, especially for the treatment of drug-resistant tumors, is an urgent need. Hence, the following drugs can inhibit tumor growth by inducing pyroptosis, providing a new idea for clinical treatment and a research direction for determining the mechanism of cancer treatment (Table 1).

**Table 1:** Drugs associated with pyroptosis

Drugs associated with pyroptosis			
Drugs	Targets	Mechanisms of pyroptosis	Cancer
Metformin	GSDMD	Degrades GSDMD, releases IL-18/1β	Oesophageal carcinoma, Hepatocellular carcinoma
	GSDME	Activates caspase-3, degrades GSDME, releases IL-18/1β	Breast cancer, Colon cancer
Arsenic	GSDME	Activates caspase-3, degrades GSDME, releases IL-18/1β	Breast cancer
Cisplatin	GSDMD	Activates NLRP3 and caspase-1, degrades GSDMD, releases IL-18/1β	Breast cancer,
Lobaplatin	GSDME	Activates caspase-3, degrades GSDME, releases IL-18/1β	Colon cancer
Dihydroartemisinin	GSDME	Activates caspase-3, degrades GSDME, releases IL-18/1β	Breast cancer, Oesophageal carcinoma
Triptolide	GSDME	Activates caspase-3, degrades GSDME, releases IL-18/1β	Neck cancer
Nobiletin	GSDMD	Degrades GSDMD and GSDME	Ovarian cancer
	GSDME		
Famotidine	GSDME	Activates GSDME, releases IL-18	Gastric cancer
Doxorubicin	GSDME	Activates caspase-3, degrades GSDME	Breast cancer
Simvastatin		Activates caspase-1 and NLRP3, releases IL-18/1β	Non-small cell lung cancer
		Inhibits caspase-1and NLRP3, the release of IL-18/1β	Glioblastoma
Cucurbitacin B	GSDMD	Activates NLRP3 and caspase-1, degrades GSDMD, releases IL-18/1β	Non-small cell lung cancer

### Doxorubicin

Doxorubicin is a common chemotherapy drug. In breast cancer (BC), GSDME exerts a certain effect on doxorubicin-mediated pyroptosis, regulating caspase-3-mediated ROS level increases and stimulating JNK phosphorylation-based activation. After doxorubicin treatment, the expression of the classical genes NLRP3 and IL-1β exhibits nonsignificant change, but the expression of caspase-3, -7, and -8 genes is increased significantly. Doxorubicin treatment induces ROS accumulation and enhances JNK phosphorylation, thereby activating the critical regulatory factor caspase-3 through cascade reactions. Moreover, doxorubicin-activated ROS play roles in caspase-8 cleavage. That is, c-caspase-8 promotes caspase-3 cleavage, and c-caspase-3 induces GSDME cleavage while inducing BC cell pyroptosis (Z. Zhang et al. 2021).

**Fig. 2:**
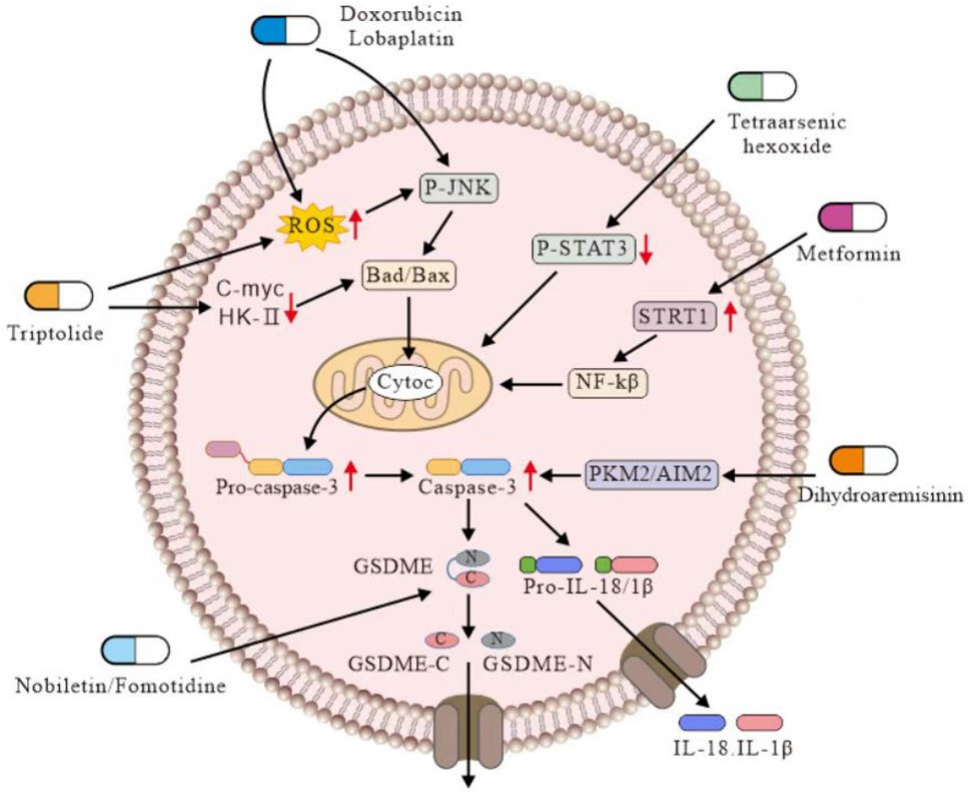
Doxorubicin and lobaplatin regulating caspase-3-mediated ROS increase and stimulating JNK phosphorylation activation-mediated pyroptosis. Dihydroartemisinin induces pyroptosis by activating cleaved caspase-3 and GSDME-N. TPL induces Bax/Bad complex translocation to mitochondria by inhibiting mitochondrial hexokinase II (HK-II) and c-myc levels, thereby releasing cyt-C into the cytoplasm while cleaving GSDME. Nobiletin and fomotidine can cleave GSDME to GSDME-C/N. Tetraarsenic hexoxide enhances mitochondrial STAT3 generation through the suppression of mitochondrial ROS phosphorylation, thereby inducing GSDME cleavage-dependent on caspase-3. Metformin induces the expression of nuclear factor-kappa B by activating SIRT1 leading to IL-1 production, triggering Bax accumulation, and releasing caspase-3 and cyt-C. All these cleave GSDME-N and lead to pyroptosis

In melanoma cells with high DFNA5 expression, doxorubicin phosphorylates the autophagy regulator eEF-2 K and cleaves DFNA5 through caspase-3 to increase lactate dehydrogenase (LDH) release, leading to pyroptosis (P. Yu et al. 2019a, b). The therapeutic dose of doxorubicin is often insufficient to cause systemic toxicity. Nonetheless, high-dose doxorubicin is not recommended due to its toxicity, particularly its cardiotoxicity (Hanušová et al. 2011). Moreover, doxorubicin induces human renal tubular epithelial cell pyroptosis through the ROS/JNK/caspase-3/GSDME signaling pathway (Fig. 2). Targeted GSDME therapy effectively reduces the nephrotoxicity induced by chemotherapeutic drugs; therefore, it can be used to reduce side effects caused by clinical treatments (Shen et al. 2021a, b).

### Arsenic

**Fig. 3:**
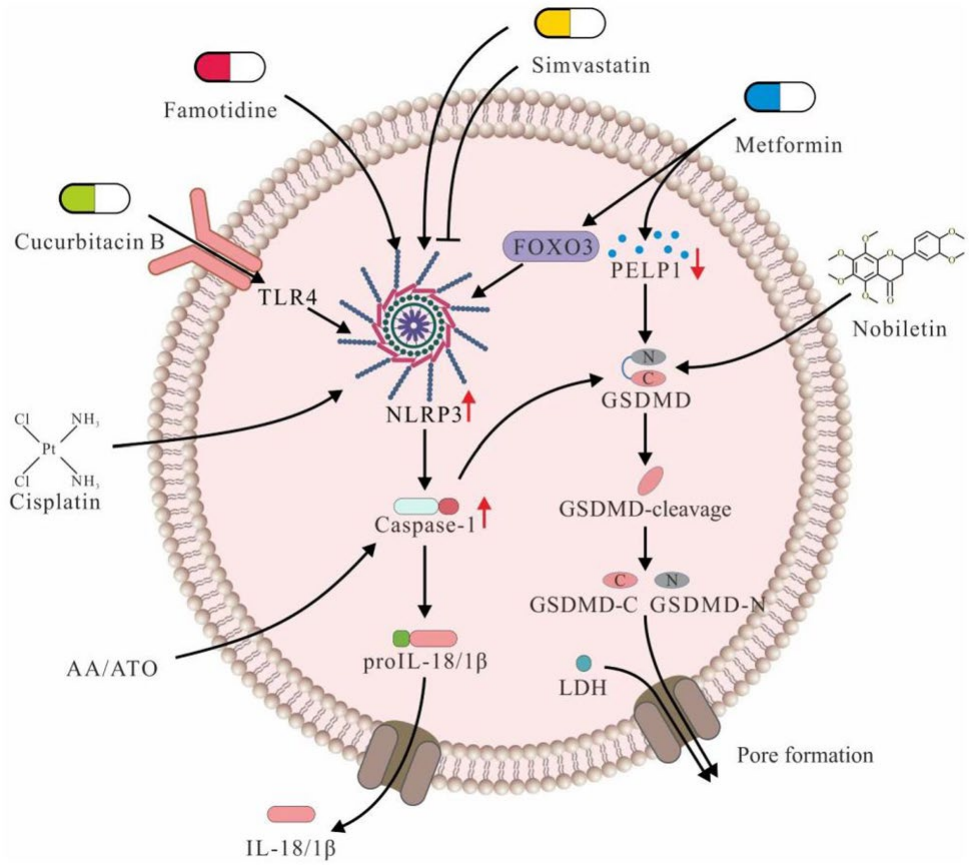
Arsenic trioxide (ATO) and ascorbic acid (AA) activate caspase-3 to trigger apoptosis, promote inflammasome formation, and induce pyroptosis. Cisplatin induces pyroptosis by upregulating the maternally expressed gene of lncRNA and inducing NLRP3/caspase-1/GSDMD molecular protein. Metformin upregulating miR-497 downregulating PELP1-induced GSDMD cleavage and upregulating FOXO3 activate NLRP3-induced pyroptosis. Simvastatin can induce caspase-1 expression and activation, increase IL-18 and IL-1β release, and induce cell pyroptosis, and also inhibit pyroptosis. CuB directly interacts with TLR4, activates NLRP3 inflammasome, increases mitochondrial ROS production, and enhances Ca^2+^ and Tom20 accumulation, thereby promoting pyroptosis. Nobiletin can induce cleaved GSDMD to GSDMD-C/N. Cisplatin can activate NLRP3-induced pyroptosis

Arsenic compounds include a number of components, among which arsenic trioxide (As2O3) and tetraarsenic hexoxide (As4O6, TetraAS) are utilized as monotherapies to treat acute promyelocytic leukemia. Acute myeloid leukemia that targets PML/RARA oncogene is rare (L. Chen et al. 2021). Arsenic trioxide (ATO) and ascorbic acid (AA) activate caspase-3 to trigger apoptosis, promote inflammasome formation, and induce pyroptosis. Additionally, ROS overexpression is the subcellular mechanism of ATO-AA induction of pyroptosis and apoptosis combined (Tian et al. 2020) (Fig. 3). Tetraarsenic hexoxide induced pyroptosis characteristics, such as balloon-like bubbling, cell swelling, and LDH release, in triple-negative breast cancer cells via pore formation in the plasma membrane, leading to caspase-3 and poly-ADP-ribose polymerase (PARP) cleavage. PARP expression was increased but did not affect normal mammary epithelial cells and did not change the difference in caspase-1 and GSDMD expression when treated with either drug. The morphology of the cells showed pyroptosis and apoptosis morphological changes, and caspase-3/PARP/GSDME cleavage occurred at the protein level. Mechanistically, pyroptosis and apoptosis play the same role and are regulated by caspase-3. When the caspase-3 inhibitor AC-devd-Cho was used, apoptosis and pyroptosis were simultaneously inhibited. However, GSDME was knocked out, the difference in caspase-3 expression was not affected while the feature of pyroptosis was significantly inhibited, suggesting that the feature of pyroptosis was generated only when caspase-3 cleaved GSDME. When observing the ultrafine structures and mechanisms of mitochondria under the effect of drugs, tetraarsenic hexoxide was found to have significantly inhibited the expression of cytochrome c in mitochondria, resulting in mitochondrial depolarization, which is related to ROS production. It also enhances mitochondrial STAT3 generation through the suppression of mitochondrial ROS phosphorylation, inducing caspase-3 dependent GSDME cleavage (An et al. 2021) (Fig. 2).

### Cisplatin and lobaplatin

Cisplatin exhibits anti-cancer activity through a variety of mechanisms. The most widely acknowledged mechanism involves its interaction with purine bases in DNA to produce DNA damage, activating multiple signal transduction pathways and ultimately leading to apoptosis. In MDA-MB-231 (BC), cisplatin induces pyroptosis by upregulating a maternally expressed lncRNA gene and inducing NLRP3/caspase-1/GSDMD molecular proteins (Fig. 3). Cisplatin activates the NLRP3 inflammasome, leading to the cleavage and activation of caspase-1. Subsequently, it cleaves GSDMD; lysed GSDMD-N fragments are released and form membrane pores to promote pyroptosis. Moreover, after activation, caspase-1 enhances pro-inflammatory factor (IL-1β or IL-18) release and maturation (Yan et al. 2021).

Lobaplatin induces ROS production and JNK phosphorylation in colon cancer cells. Activated JNK recruits BAX to mitochondria, thereby stimulating cyt-c production in the cytoplasm; subsequently, GSDME cleavage via caspase-3/-9 induces pyroptosis. Thus, GSDME mediates pyroptosis after caspase-3/-9 activation in the lobaplatin-induced downstream ROS/JNK/BAX-mitochondrial apoptotic pathway in colon cancer cells (Fig. 2). In nasopharyngeal carcinoma, lobaplatin induces cell death and suppresses tumor cell proliferation by modulating cIAP1/2 and ribosome activity and ROS production (Z. Chen et al. 2020).

### Metformin

Metformin is a first-line clinical drug used to treat type-2 diabetes mellitus. It inhibits gluconeogenesis by blocking the mitochondrial redox shuttle in the liver. A study revealed that patients taking metformin for diabetes may exhibit a lower incidence of pancreatic cancer (Flory & Lipska 2019; LaMoia & Shulman 2021; H. Yu et al. 2019a, b). Metformin may play a role in tumorigenesis by inducing autophagy after passing through the blood‒brain barrier or in ferroptosis through the UFMylation of SLC7A11, thereby suppressing tumor metastasis and invasion (Lu et al. 2021; J. Yang et al. 2021). PELP1 is highly expressed in esophageal squamous cell carcinoma (ESCC). The significance of an increase in the PELP1 level in tumor recurrence and prognosis is especially obvious in patients with advanced ESCC. Metformin led to pyroptosis in ESCC, inducing pyroptosis by upregulating miR-497 activity and downregulating PELP1-induced GSDMD cleavage, causing cells to undergo pyroptosis. In hepatocellular carcinoma cells, metformin led to pyroptosis, upregulated FOXO3-activated NLRP3 transcription, and promoted IL-1β/IL-18, NLRP3, and c-caspase-1 level increases (Z. Shen et al. 2021a, b; L. Wang et al. 2019) (Fig. 3). In addition, metformin induced the expression of nuclear factor-kappa B (NF-κB) by activating SIRT1 in colorectal cancer and breast cancer cells, leading to IL-1β production, triggering BAX accumulation, and releasing caspase-3 and cyt-C. All these events led to GSDME-N cleavage and pyroptosis (Zheng et al. 2020) (Fig. 2).

### Dihydroartemisinin

Dihydroartemisinin (DHA) is a derivative of artemisinin and is a major drug used to treat malaria (Gutman et al. 2017). Studies have demonstrated the inhibitory effects of DHA on tumor cell proliferation and metastasis and tumor angiogenesis, as well as its enhancing effect on immunity (Dai et al. 2021). DHA kills tumor cells by inducing ROS production to produce oxidative stress, blocking the cell cycle, and inducing cell death. It promotes the anti-cancer immune response and ferroptosis (Y. Chen et al. 2019). In BC cells, DHA promoted the release of LDH and reduced the amount of ATP released. DHA increased IL-1β, IL-18, caspase-3, AIM2, and HMGB1 levels; moreover, DHA cleaved DFNA5-FL into DFNA5-N fragments to cause pyroptosis. AIM2 is an inflammasome associated with pyroptosis. When AIM2 was knocked down, cell invasiveness and migratory capacity were significantly inhibited, and the expression of GSDME and caspase-3 was inhibited, IL-1β and IL-18 are also inhibited in ELISA test (Y. Li et al. 2021a, b). In ESCC (Eca109 and Ec9706) treated with DHA, significant pyroptosis features, such as chromatin fixation, cytoplasmic loss, and endoplasmic reticulum swelling, were evident. DHA downregulated the expression of the pyruvate kinase isoform M2 (PKM2, an important glycolytic enzyme), promoted caspase-3/-8 activation, accelerated GSDME cleavage to generate GSDME-N, and increased LDH levels and inflammatory cytokine (IL-1β, IL-18) production. However, when a caspase-3 inhibitor (Ac-DEVD-CHO) was used to treat cells, the inhibition of cell growth induced by DHA was significantly attenuated, and the amount of LDH and IL-18/IL-1β released was also decreased. When the expression of GSDME was silenced, the pyroptosis state of the cells was significantly reversed, and no significant change in caspase-3 level compared to that in the control group was found, indicating that GSDME was located downstream of caspase-3. However, DHA did not activate GSDMD or caspase-1 directly. In fact, it induced pyroptosis by activating cleaved caspase-3 and GSDME-N (Jiang et al. 2021) (Fig. 2).

### Triptolide

Triptolide (TPL) is a diterpenoid tricyclic oxide extracted from *Tripterygium wilfordii* roots and exhibits anti-inflammatory, anti-cancer, and anti-immunosuppressive effects. It also mitigates bowel inflammation by protecting the intestine by suppressing M1 polarization and macrophage infiltration, and inhibiting inflammatory factor expression (Tang et al. 2020). In pancreatic cancer cells, TPL decreased NF-κB signal transduction (the main EMT pathway) and reduced the expression of related transcription factors, such as ZEB1, SNAI1, and SNAI2, with mesenchymal markers, such as vimentin and CDH2 (Noel et al. 2019).

From the pyroptosis perspective, in the HK1 and FaDu squamous nasopharyngeal carcinoma cell lines with high GSDME expression, TPL induced pyroptosis cell lines, but in the C666-1 cell line with low GSDME expression, TPL did not induce pyroptosis reactions, such as an increase in the release of LDH or IL-1β, indicating that TPL induced pyroptosis by cleaving GSDME. Silencing the expression of GSDME in cell lines prevented TPL-induced cell death, decreased cell mortality and reduced the rate of LDH and IL-1β release. During the induction of pyroptosis, the expression of BAX, BAD and caspase-3 increased in HK1 and FaDu cells but not in C666-1 cells. When BAX and BAD were silenced, the expression of caspase-3 and GSDME decreased. In addition, the use of the caspase inhibitor z-VAD inhibited the expression of GSDME. The results showed that activation of the BAD/BAX-caspase3 cascade was necessary for GSDME cleavage and pyroptosis. TPL induced BAD/BAX complex translocation to mitochondria by reducing mitochondrial hexokinase II (HK-II) and c-myc levels, triggering the release of cyt-c into the cytoplasm while GSDME was cleaved. Silencing HK-II did not promote pyroptosis but promoted TPL-induced pyroptosis, caspase-3 expression and GSDME cleavage. However, when the GSDME level was high, the pyroptosis reaction was inhibited, and the translocation ability of the BAD and BAX proteins was reduced (Fig. 2). TPL exerts a critical effect on the anticancer immune response. TPL also suppresses cancer cell proliferation by inhibiting the NRF2/SLC7A11 axis and inducing ROS accumulation (Cai et al. 2021).

### Nobiletin

Nobiletin is a polyethoxy flavonoid found in citrus fruits and exerts several favorable pharmacological and biological effects against inflammation, tumors, and non-alcoholic fatty liver (Li et al. 2022; Nohara et al. 2019; H. Wang et al. 2020). In A2780 and OVCAR3 ovarian cancer cells, nobiletin induces ROS release and alters mitochondrial membrane potential and the cleavage of GSDMD and GSDME. When using the ROS inhibitor N-acetylcysteine (NAC), the cleanliness of GSDMD and GSDME was obviously reversed (Fig. 2). NAC reversed the expression of LC3-II, the production of ROS is caused by autophagy. The autophagy inhibitor 3-MA not only inhibited the production of LC3-II but also reversed the increase in GSDMD and GSDME cleavage. Therefore, it can be inferred that nobiletin leads to pyroptosis mediated through autophagy in ovarian cancer cells (R. Zhang et al. 2020). In BC, nobiletin increased GSDMD and NLRP3 expression. A miR-200b simulator enhanced the effect of nobiletin and induced pyroptosis. These findings suggest that nobiletin promotes the pyroptosis of breast cells by regulating the miR-200b/JAZF1 axis in the presence of NF-κ B (J. G. Wang et al. 2021a, b) (Fig. 3).

### Famotidine

Famotidine plays a certain role in neuritis, gastric ulcer, duodenal ulcer, and heterotopic ossification (Unal, Dokumaci, Ozkartal, Yerer, & Aricioglu, 2019; Yamamoto et al. 2012). Famotidine prevents ischemia reperfusion injury by inhibiting the increase in malonaldehyde and NO, markers of oxidative stress, and increasing the production of antioxidants (Tanriverdi et al. 2021). A study of colorectal cancer revealed that preoperative use of famotidine increased the ability of lymphocytes to infiltrate tumors and inhibited tumor development (Kapoor et al. 2005). However, we found that famotidine activated the NLPR3 inflammasome in gastric carcinoma cell to induce the production of ASC and caspase-1, thereby inducing IL-18 release and maturation, which were associated with a famotidine-induced increase in ERK1/2 phosphorylation levels. Famotidine reduced ERK1/2 expression by inhibiting IL-18 phosphorylation. Inhibition of ERK1/2 activity abolished the famotidine effect on IL-18 and LDH levels, indicating a role for famotidine in regulating ERK1/2-dependent cell death. Moreover, famotidine induced pyroptosis by inducing GSDME cleavage; however, it exerted no effect on GSDMD (Huang et al. 2021) (Fig. 3).

### Simvastatin

Simvastatin is the hydroxymethyl glutaryl coenzyme A (HMG-COA) reductase inhibitor, which inhibits the synthesis of endogenous cholesterol and decreases the levels of circulatory lipids, especially low-density lipoprotein cholesterol (Duarte et al. 2021). It also exhibits neuroprotective effects by reducing inflammation, caspase-3 activation, and apoptosis to reduce cerebral hypoxia–ischemia injury in rat neonates (Carloni et al. 2006; Menze et al. 2021). Simvastatin can inhibit the proliferation and migration of tumor cells through oxidative stress and DNA damage (Jaqueline Aparecida Duarte, Andre Luis Branco de Barros, & Elaine Amaral Leite, 2021). In non-small cell lung cancer (NSCLC), simvastatin induced caspase-1 expression and activation, increases IL-18 and IL-1β release, and induced NSCLC cell pyroptosis (F. Wang et al. 2018). However, in U87 and U251 glioma cells, simvastatin inhibited pyroptosis and caused cell death by inhibiting caspase-1, NLRP3, and IL-1β production. MiR-214–3p partially inhibited glioma cell growth and invasion by regulating caspase-1-mediated pyroptosis. When miR-214-3p is silenced, it promotes glioma cell growth. These findings indicate that simvastatin exerts its inhibitory effect through apoptosis induced by caspase-1, and miR-214–3p is a possible target by which simvastatin regulates tumor cell growth (S. Yang et al. 2022) (Fig. 3).

### Cucurbitacin B

Cucurbitacin B (CuB), obtained from muskmelon pedicel (Yuan et al. 2021), exhibits several pharmacological effects, such as anti-inflammatory, anti-oxidative, anti-viral, anti-pyretic, analgesic, and anti-malarial activity (Kim et al. 2015; Park, Kim; Park 2015; Xiao et al. 2017). CuB inhibited BC growth and induced the arrest of the cell cycle at G2/M, leading to cell death (Garg et al. 2018). CuB directly interacts with TLR4, activates the NLRP3 inflammasome, increases mitochondrial ROS production, and enhances Ca^2+^ and Tom20 accumulation, thereby promoting pyroptosis. TLR4 is an activation signaling molecule upstream of the NLRP3 inflammasome and promotes pyroptosis in several cell types. TLR4 interacts with MyD88 (downstream partner) on the cell membrane, inducing cytokine and ROS generation. In addition, TLR4 induces caspase-1 activation, resulting in inflammatory factor release and cell death. After CuB treatment, the TLR4 protein level is increased. CuB suppressed NSCLC growth both in vitro and in vivo by promoting TLR4/NLRP3/GSDMD mediated pyroptosis (Fig. 3). According to these findings, CuB directly interacts with TLR4 to activate the NLRP3 inflammasome while increasing mitochondrial ROS production and Ca^2+^ and Tom20 accumulation, thereby promoting pyroptosis (Yuan et al. 2021).

## Conclusions

Pyroptosis is a form of inflammatory cell death, and several drugs induce pyroptosis to suppress cancer cell proliferation. Although its effectiveness and adverse reactions are highly pertinent to patients receiving clinical treatment, its potential use in cancer treatment cannot be ruled out. In terms of cancer, cell death seems to be a double-edged sword. Tumor cell pyroptosis can be leveraged as a therapeutic target for inhibiting tumorigenesis and progression. However, healthy cells release inflammatory cytokines during pyroptosis, leading to the formation of an inflammatory microenvironment and the transformation of healthy cells into tumor cells (Xia et al. 2019). Moreover, pyroptosis enhances tumor immunogenicity by attracting an increased number antitumor lymphocytes that turn “cold” tumors into “hot” tumors, thereby enhancing host tumor immunity. Through deeper research on the relationship between drugs and genes, we can identify effective targets. A member of the gasdermin family may be a marker of cancer prognosis, a possibility requiring further research. At present, we have conducted only in vitro experiments. However, we should explore the side effects of drug candidates in clinical settings. Pyroptosis pathogenesis needs to be further studied, and an in-depth understanding of pyroptosis will certainly benefit the treatment of related diseases. The use of drugs provides a good research basis for clinical treatment, but the toxic effects of drugs are also worth considering. For example, with the development of nanomaterials, it is now possible to deliver drugs while reducing damage to non-targeted tissues and maximize tumor treatment efficiency. Will there be a more significant development in treatment (Wu et al. 2021a, b; M. Wu et al. 2021a, b; Zhao et al. 2020)? To answer this question, further consideration and study are worthwhile.

## Data Availability

Not applicable.

## Abbreviations

PFD: Pore-forming domain
RD: Repression domain
BC: Breast cancer
ESCC: Epidemiology of esophageal squamous cell carcinoma
PRR: Pattern-recognition receptors
PAMPs: Pathogen-associated molecular patterns
DAMPs: Damage-associated molecular patterns
TLRs: Toll-like receptors
RLRs: Retinoic acid-induced gene I (RIG-I)-like receptors
NLRs: Nucleotide oligomerization domain (NOD)-like receptors
NLRP3: NOD-like receptor protein 3
PYD: Pyrin domain
IL-18: Interleukin-18
IL-1β: Interleukin-1β
ROS: Reactive oxygen species
CARD: Crystal structure of caspase recruiting domain
ASC: Apoptosis-associated speck-like protein containing a CARD
LPS: Lipopolysaccharide
GSDMD: Gasdermin D
GSDME: Gasdermin E
LDH: Lactate dehydrogenase
ATP: Adenosine triphosphate

## References

[CR1] Alu A, Han X, Ma X, Wu M, Wei Y, Wei X (2020). The role of lysosome in regulated necrosis. Acta Pharm Sin B.

[CR2] An H, Heo JS, Kim P, Lian Z, Lee S, Park J, Yang KM (2021). Tetraarsenic hexoxide enhances generation of mitochondrial ROS to promote pyroptosis by inducing the activation of caspase-3/GSDME in triple-negative breast cancer cells. Cell Death Dis.

[CR3] Bergsbaken T, Fink SL, Cookson BT (2009). Pyroptosis: host cell death and inflammation. Nat Rev Microbiol.

[CR4] Burdette BE, Esparza AN, Zhu H, Wang S (2021). Gasdermin D in pyroptosis. Acta Pharm Sin B.

[CR5] Burgener SS, Schroder K (2020). Neutrophil Extracellular Traps in Host Defense. Cold Spring Harb Perspect Biol.

[CR6] Cai J, Yi M, Tan Y, Li X, Li G, Zeng Z, Xiang B (2021). Natural product triptolide induces GSDME-mediated pyroptosis in head and neck cancer through suppressing mitochondrial hexokinase-ΙΙ. J Exp Clin Cancer Res.

[CR7] Carloni S, Mazzoni E, Cimino M, De Simoni MG, Perego C, Scopa C, Balduini W (2006). Simvastatin reduces caspase-3 activation and inflammatory markers induced by hypoxia-ischemia in the newborn rat. Neurobiol Dis.

[CR8] Chen Y, Mi Y, Zhang X, Ma Q, Song Y, Zhang L, Zou Z (2019). Dihydroartemisinin-induced unfolded protein response feedback attenuates ferroptosis via PERK/ATF4/HSPA5 pathway in glioma cells. J Exp Clin Cancer Res.

[CR9] Chen Z, Xu G, Wu D, Wu S, Gong L, Li Z, Li X (2020). Lobaplatin induces pyroptosis through regulating cIAP1/2, Ripoptosome and ROS in nasopharyngeal carcinoma. Biochem Pharmacol.

[CR10] Chen L, Zhu HM, Li Y, Liu QF, Hu Y, Zhou JF, Li JM (2021). Arsenic trioxide replacing or reducing chemotherapy in consolidation therapy for acute promyelocytic leukemia (APL2012 trial). Proc Natl Acad Sci U S A.

[CR11] Cookson BT, Brennan MA (2001). Pro-inflammatory programmed cell death. Trends Microbiol.

[CR12] Dai X, Zhang X, Chen W, Chen Y, Zhang Q, Mo S, Lu J (2021). Dihydroartemisinin: A Potential Natural Anticancer Drug. Int J Biol Sci.

[CR13] Ding J, Wang K, Liu W, She Y, Sun Q, Shi J, Shao F (2016). Pore-forming activity and structural autoinhibition of the gasdermin family. Nature.

[CR14] Duarte JA, de Barros ALB, Leite EA (2021). The potential use of simvastatin for cancer treatment: A review. Biomed Pharmacother.

[CR15] Fang Y, Tian S, Pan Y, Li W, Wang Q, Tang Y, Shu Y (2020). Pyroptosis: A new frontier in cancer. Biomed Pharmacother.

[CR16] Fernandes-Alnemri T, Wu J, Yu JW, Datta P, Miller B, Jankowski W, Alnemri ES (2007). The pyroptosome: a supramolecular assembly of ASC dimers mediating inflammatory cell death via caspase-1 activation. Cell Death Differ.

[CR17] Flory J, Lipska K (2019). Metformin in 2019. JAMA.

[CR18] Galluzzi L, Vitale I, Aaronson SA, Abrams JM, Adam D, Agostinis P, Kroemer G (2018). Molecular mechanisms of cell death: recommendations of the Nomenclature Committee on Cell Death 2018. Cell Death Differ.

[CR19] Garg S, Kaul SC, Wadhwa R (2018). Cucurbitacin B and cancer intervention: Chemistry, biology and mechanisms (Review). Int J Oncol.

[CR20] Guo H, Callaway JB, Ting JP (2015). Inflammasomes: mechanism of action, role in disease, and therapeutics. Nat Med.

[CR21] Gutman J, Kovacs S, Dorsey G, Stergachis A, Ter Kuile FO (2017). Safety, tolerability, and efficacy of repeated doses of dihydroartemisinin-piperaquine for prevention and treatment of malaria: a systematic review and meta-analysis. Lancet Infect Dis.

[CR22] Hanušová V, Boušová I, Skálová L (2011). Possibilities to increase the effectiveness of doxorubicin in cancer cells killing. Drug Metab Rev.

[CR23] He WT, Wan H, Hu L, Chen P, Wang X, Huang Z, Han J (2015). Gasdermin D is an executor of pyroptosis and required for interleukin-1β secretion. Cell Res.

[CR24] Huang J, Fan P, Liu M, Weng C, Fan G, Zhang T, Liu Y (2021). Famotidine promotes inflammation by triggering cell pyroptosis in gastric cancer cells. BMC Pharmacol Toxicol.

[CR25] Jiang M, Wu Y, Qi L, Li L, Song D, Gan J, Song C (2021). Dihydroartemisinin mediating PKM2-caspase-8/3-GSDME axis for pyroptosis in esophageal squamous cell carcinoma. Chem Biol Interact.

[CR26] Jorgensen I, Miao EA (2015). Pyroptotic cell death defends against intracellular pathogens. Immunol Rev.

[CR27] Kapoor S, Pal S, Sahni P, Dattagupta S, Kanti Chattopadhyay T (2005). Effect of pre-operative short course famotidine on tumor infiltrating lymphocytes in colorectal cancer: a double blind, placebo controlled, prospective randomized study. J Surg Res.

[CR28] Kayagaki N, Stowe IB, Lee BL, O'Rourke K, Anderson K, Warming S, Dixit VM (2015). Caspase-11 cleaves gasdermin D for non-canonical inflammasome signalling. Nature.

[CR29] Kelley N, Jeltema D, Duan Y, He Y (2019). The NLRP3 inflammasome: an overview of mechanisms of activation and regulation. Int J Mol Sci.

[CR30] Kim M, Park SY, Jin ML, Park G, Son HJ (2015). Cucurbitacin B inhibits immunomodulatory function and the inflammatory response in macrophages. Immunopharmacol Immunotoxicol.

[CR31] Kovacs SB, Miao EA (2017). Gasdermins: Effectors of Pyroptosis. Trends Cell Biol.

[CR32] Kuang S, Zheng J, Yang H, Li S, Duan S, Shen Y, Li J (2017). Structure insight of GSDMD reveals the basis of GSDMD autoinhibition in cell pyroptosis. Proc Natl Acad Sci U S A.

[CR33] Lamkanfi M, Dixit VM (2017). In Retrospect: The inflammasome turns 15. Nature.

[CR34] LaMoia TE, Shulman GI (2021). Cellular and Molecular Mechanisms of Metformin Action. Endocr Rev.

[CR35] Li L, Jiang M, Qi L, Wu Y, Song D, Gan J, Bai Y (2021). Pyroptosis, a new bridge to tumor immunity. Cancer Sci.

[CR36] Li Y, Wang W, Li A, Huang W, Chen S, Han F, Wang L (2021). Dihydroartemisinin induces pyroptosis by promoting the AIM2/caspase-3/DFNA5 axis in breast cancer cells. Chem Biol Interact.

[CR37] Li S, Li X, Chen F, Liu M, Ning L, Yan Y, Tu C (2022). Nobiletin mitigates hepatocytes death, liver inflammation, and fibrosis in a murine model of NASH through modulating hepatic oxidative stress and mitochondrial dysfunction. J Nutr Biochem.

[CR38] Liu X, Zhang Z, Ruan J, Pan Y, Magupalli VG, Wu H, Lieberman J (2016). Inflammasome-activated gasdermin D causes pyroptosis by forming membrane pores. Nature.

[CR39] Liu Z, Wang C, Yang J, Zhou B, Yang R, Ramachandran R, Xiao TS (2019). Crystal structures of the full-length murine and human gasdermin d reveal mechanisms of autoinhibition, lipid binding, and oligomerization. Immunity.

[CR40] Loveless R, Bloomquist R, Teng Y (2021). Pyroptosis at the forefront of anticancer immunity. J Exp Clin Cancer Res.

[CR41] Lu G, Wu Z, Shang J, Xie Z, Chen C, Zhang C (2021). The effects of metformin on autophagy. Biomed Pharmacother.

[CR42] Martinon F, Burns K, Tschopp J (2002). The inflammasome: a molecular platform triggering activation of inflammatory caspases and processing of proIL-beta. Mol Cell.

[CR43] Massagué J, Obenauf AC (2016). Metastatic colonization by circulating tumour cells. Nature.

[CR44] Menze ET, Ezzat H, Shawky S, Sami M, Selim EH, Ahmed S, Michel HE (2021). Simvastatin mitigates depressive-like behavior in ovariectomized rats: Possible role of NLRP3 inflammasome and estrogen receptors' modulation. Int Immunopharmacol.

[CR45] Morrison AH, Byrne KT, Vonderheide RH (2018). Immunotherapy and Prevention of Pancreatic Cancer. Trends Cancer.

[CR46] Noel P, Von Hoff DD, Saluja AK, Velagapudi M, Borazanci E, Han H (2019). Triptolide and Its Derivatives as Cancer Therapies. Trends Pharmacol Sci.

[CR47] Nohara K, Mallampalli V, Nemkov T, Wirianto M, Yang J, Ye Y, Chen Z (2019). Nobiletin fortifies mitochondrial respiration in skeletal muscle to promote healthy aging against metabolic challenge. Nat Commun.

[CR48] Park SY, Kim YH, Park G (2015). Cucurbitacins attenuate microglial activation and protect from neuroinflammatory injury through Nrf2/ARE activation and STAT/NF-κB inhibition. Neurosci Lett.

[CR49] Rathinam VAK, Zhao Y, Shao F (2019). Innate immunity to intracellular LPS. Nat Immunol.

[CR50] Rogers C, Fernandes-Alnemri T, Mayes L, Alnemri D, Cingolani G, Alnemri ES (2017). Cleavage of DFNA5 by caspase-3 during apoptosis mediates progression to secondary necrotic/pyroptotic cell death. Nat Commun.

[CR51] Ruan J, Wang S, Wang J (2020). Mechanism and regulation of pyroptosis-mediated in cancer cell death. Chem Biol Interact.

[CR52] Sborgi L, Rühl S, Mulvihill E, Pipercevic J, Heilig R, Stahlberg H, Hiller S (2016). GSDMD membrane pore formation constitutes the mechanism of pyroptotic cell death. Embo j.

[CR53] Shen X, Wang H, Weng C, Jiang H, Chen J (2021). Caspase 3/GSDME-dependent pyroptosis contributes to chemotherapy drug-induced nephrotoxicity. Cell Death Dis.

[CR54] Shen Z, Zhou H, Li A, Wu T, Ji X, Guo L, He X (2021). Metformin inhibits hepatocellular carcinoma development by inducing apoptosis and pyroptosis through regulating FOXO3. Aging (albany NY).

[CR55] Shi J, Zhao Y, Wang K, Shi X, Wang Y, Huang H, Shao F (2015). Cleavage of GSDMD by inflammatory caspases determines pyroptotic cell death. Nature.

[CR56] Shi J, Gao W, Shao F (2017). Pyroptosis: Gasdermin-Mediated Programmed Necrotic Cell Death. Trends Biochem Sci.

[CR57] Silke J, Vince J (2017). IAPs and Cell Death. Curr Top Microbiol Immunol.

[CR58] Tan Y, Chen Q, Li X, Zeng Z, Xiong W, Li G, Yi M (2021). Pyroptosis: a new paradigm of cell death for fighting against cancer. J Exp Clin Cancer Res.

[CR59] Tang B, Zhu J, Zhang B, Wu F, Wang Y, Weng Q, Ji J (2020). Therapeutic Potential of Triptolide as an Anti-Inflammatory Agent in Dextran Sulfate Sodium-Induced Murine Experimental Colitis. Front Immunol.

[CR60] Tanriverdi HI, Şenel U, Gevrek F, Akbaş A (2021). Protective effect of famotidine on ischemia-reperfusion injury following testicular torsion in rats. J Pediatr Urol.

[CR61] Tian W, Wang Z, Tang NN, Li JT, Liu Y, Chu WF, Yang BF (2020). Ascorbic Acid Sensitizes Colorectal Carcinoma to the Cytotoxicity of Arsenic Trioxide via Promoting Reactive Oxygen Species-Dependent Apoptosis and Pyroptosis. Front Pharmacol.

[CR62] Torii S, Shintoku R, Kubota C, Yaegashi M, Torii R, Sasaki M, Yamada K (2016). An essential role for functional lysosomes in ferroptosis of cancer cells. Biochem J.

[CR63] Unal G, Dokumaci AH, Ozkartal CS, Yerer MB, Aricioglu F (2019). Famotidine has a neuroprotective effect on MK-801 induced toxicity via the Akt/GSK-3β/β-catenin signaling pathway in the SH-SY5Y cell line. Chem Biol Interact.

[CR64] Van Gorp H, Lamkanfi M (2019). The emerging roles of inflammasome-dependent cytokines in cancer development. EMBO Rep.

[CR65] Wang Y, Gao W, Shi X, Ding J, Liu W, He H, Shao F (2017). Chemotherapy drugs induce pyroptosis through caspase-3 cleavage of a gasdermin. Nature.

[CR66] Wang F, Liu W, Ning J, Wang J, Lang Y, Jin X, Xu S (2018). Simvastatin Suppresses Proliferation and Migration in Non-small Cell Lung Cancer via Pyroptosis. Int J Biol Sci.

[CR67] Wang L, Li K, Lin X, Yao Z, Wang S, Xiong X, Zhang H (2019). Metformin induces human esophageal carcinoma cell pyroptosis by targeting the miR-497/PELP1 axis. Cancer Lett.

[CR68] Wang H, Guo Y, Qiao Y, Zhang J, Jiang P (2020). Nobiletin Ameliorates NLRP3 Inflammasome-Mediated Inflammation Through Promoting Autophagy via the AMPK Pathway. Mol Neurobiol.

[CR69] Wang JG, Jian WJ, Li Y, Zhang J (2021). Nobiletin promotes the pyroptosis of breast cancer via regulation of miR-200b/JAZF1 axis. Kaoh J Med Sci.

[CR70] Wang L, Sharif H, Vora SM, Zheng Y, Wu H (2021). Structures and functions of the inflammasome engine. J Allergy Clin Immunol.

[CR71] Wu D, Wang S, Yu G, Chen X (2021). Cell Death Mediated by the Pyroptosis Pathway with the Aid of Nanotechnology: Prospects for Cancer Therapy. Angew Chem Int Ed Engl.

[CR72] Wu M, Liu X, Chen H, Duan Y, Liu J, Pan Y, Liu B (2021). Activation of Pyroptosis by Membrane-Anchoring AIE Photosensitizer Design: New Prospect for Photodynamic Cancer Cell Ablation. Angew Chem Int Ed Engl.

[CR73] Xia X, Wang X, Cheng Z, Qin W, Lei L, Jiang J, Hu J (2019). The role of pyroptosis in cancer: pro-cancer or pro-"host"?. Cell Death Dis.

[CR74] Xiao Y, Yang Z, Wu QQ, Jiang XH, Yuan Y, Chang W, Tang QZ (2017). Cucurbitacin B Protects Against Pressure Overload Induced Cardiac Hypertrophy. J Cell Biochem.

[CR75] Xu YJ, Zheng L, Hu YW, Wang Q (2018). Pyroptosis and its relationship to atherosclerosis. Clin Chim Acta.

[CR76] Yamamoto K, Hojo H, Koshima I, Chung UI, Ohba S (2012). Famotidine suppresses osteogenic differentiation of tendon cells in vitro and pathological calcification of tendon in vivo. J Orthop Res.

[CR77] Yan H, Luo B, Wu X, Guan F, Yu X, Zhao L, Yuan J (2021). Cisplatin Induces Pyroptosis via Activation of MEG3/NLRP3/caspase-1/GSDMD Pathway in Triple-Negative Breast Cancer. Int J Biol Sci.

[CR78] Yang J, Zhou Y, Xie S, Wang J, Li Z, Chen L, Zhou J (2021). Metformin induces Ferroptosis by inhibiting UFMylation of SLC7A11 in breast cancer. J Exp Clin Cancer Res.

[CR79] Yang S, Xie C, Guo T, Li H, Li N, Zhou S, Xie C (2022). Simvastatin Inhibits Tumor Growth and Migration by Mediating Caspase-1-dependent Pyroptosis in Glioblastoma Multiforme. World Neurosurg.

[CR80] Yu H, Zhong X, Gao P, Shi J, Wu Z, Guo Z, Song Y (2019). The Potential Effect of Metformin on Cancer: An Umbrella Review. Frontiers Endocrinol.

[CR81] Yu P, Wang HY, Tian M, Li AX, Chen XS, Wang XL, Cheng Y (2019). Eukaryotic elongation factor-2 kinase regulates the cross-talk between autophagy and pyroptosis in doxorubicin-treated human melanoma cells in vitro. Acta Pharmacol Sin.

[CR82] Yu P, Zhang X, Liu N, Tang L, Peng C, Chen X (2021). Pyroptosis: mechanisms and diseases. Signal Transduct Target Ther.

[CR83] Yuan R, Zhao W, Wang QQ, He J, Han S, Gao H, Yang S (2021). Cucurbitacin B inhibits non-small cell lung cancer in vivo and in vitro by triggering TLR4/NLRP3/GSDMD-dependent pyroptosis. Pharmacol Res.

[CR84] Zhang R, Chen J, Mao L, Guo Y, Hao Y, Deng Y, Yuan M (2020). Nobiletin Triggers Reactive Oxygen Species-Mediated Pyroptosis through Regulating Autophagy in Ovarian Cancer Cells. J Agric Food Chem.

[CR85] Zhang Z, Zhang H, Li D, Zhou X, Qin Q, Zhang Q (2021). Caspase-3-mediated GSDME induced Pyroptosis in breast cancer cells through the ROS/JNK signalling pathway. J Cell Mol Med.

[CR86] Zhao P, Wang M, Chen M, Chen Z, Peng X, Zhou F, Qu J (2020). Programming cell pyroptosis with biomimetic nanoparticles for solid tumor immunotherapy. Biomaterials.

[CR87] Zheng Z, Bian Y, Zhang Y, Ren G, Li G (2020). Metformin activates AMPK/SIRT1/NF-κB pathway and induces mitochondrial dysfunction to drive caspase3/GSDME-mediated cancer cell pyroptosis. Cell Cycle.

